# The Association Between Vitamin B1 Deficiency and Anemia Among Elderly Patients at a Rural Hospital in Japan: A Cross-Sectional Study

**DOI:** 10.7759/cureus.47173

**Published:** 2023-10-17

**Authors:** Toshiki Fukunaga, Ryuichi Ohta, Chiaki Sano

**Affiliations:** 1 Family Medicine, Community Medicine Management, Faculty of Medicine, Shimane University, Izumo, JPN; 2 Community Care, Unnan City Hospital, Unnan, JPN; 3 Medicine, Community Medicine Management, Faculty of Medicine, Shimane University, Izumo, JPN

**Keywords:** retrospective study, neuropsychiatric symptoms, wernicke's encephalopathy, multivariate logistic regression, gastrointestinal tract, thiamine-responsive megaloblastic anemia (trma), hospitalized patients, rural japan, anemia, vitamin b1 deficiency

## Abstract

Background and objective

Vitamin B1 deficiency can cause a variety of abnormalities in the neuropsychiatric, cardiovascular, and other systems. This condition can be rapidly corrected and prevented from progressing to irreversible sequelae through vitamin B1 supplementation. Therefore, early detection of and intervention in vitamin B1 deficiency are essential. We have previously demonstrated an association between vitamin B1 deficiency and appetite loss in hospitalized older adult patients in rural Japan. This study aimed to examine the additional predictors of vitamin B1 deficiency in patients with appetite loss and other symptoms suggestive of vitamin B1 deficiency.

Material and methods

This cross-sectional study involved 519 patients admitted to a rural hospital between April 2020 and March 2022. Data on vitamin B1 levels, age, sex, BMI, albumin levels, functional independence measure (FIM), hemoglobin levels, Charlson Comorbidity Index (CCI), and medications were collected from electronic medical records. Vitamin B1 deficiency was defined as serum vitamin B1 level <20 µg/dL. Data were analyzed using the Mann-Whitney U test, Student's t-test, and chi-square test, followed by multivariate logistic regression to examine the predictors of vitamin B1 deficiency.

Results

A total of 113 patients (21.5%) were found to be vitamin B1-deficient. Multivariate logistic regression showed that anemia was significantly associated with vitamin B1 deficiency [adjusted odds ratio (AOR): 1.71, 95% confidence interval (CI): 1.07-2.73, p<0.05].

Conclusion

Based on our findings, anemia is significantly associated with vitamin B1 deficiency in hospitalized Japanese patients living in rural areas. Therefore, physicians should be mindful of the possibility of vitamin B1 deficiency in hospitalized patients with anemia.

## Introduction

Vitamin B1 is a water-soluble vitamin that acts as a coenzyme for energy production. Water-soluble vitamins are not stored in surplus in the body, leading to their deficiency if not regularly consumed in sufficient amounts. Vitamin B1 deficiency causes various symptoms in the nervous and cardiovascular systems, such as Beriberi syndrome, Wernicke's encephalopathy, and Korsakoff syndrome; in some severe cases, it can even be fatal [1,2]. On the other hand, vitamin B1 deficiency symptoms can be quickly corrected and their progression prevented by vitamin B1 supplementation [3]. Therefore, early detection of and intervention in vitamin B1 deficiency are essential.

Vitamin B1 is abundant in white rice and red meat. Their intake is likely influenced by cultural background, lifestyle, and geographical characteristics [4,5]. In rural areas of Japan, plant-based foods constitute the most common diet, and red meat consumption is low [6]. In recent years, junk food consumption has become widespread, even in rural areas, and opportunities to consume foods rich in vitamin B1 may decrease further. Additionally, vitamin B1 can be lost from food, and its absorption in the gastrointestinal tract can be weakened by cooking [7,8]. Abnormalities in the gastrointestinal mucosa can also reduce absorption. Older individuals often have multiple comorbidities, including atrophy of the gastrointestinal tract and polypharmacy [9,10]. These conditions can reduce vitamin B1 absorption and cause appetite loss. In addition to these food cultures, rural Japan has a highly aged population, presumably at high risk of vitamin B1 deficiency.

Identifying the indicators of vitamin B1 deficiency in rural Japan will aid in the early treatment of vitamin B1 deficiency and the prevention of its irreversible sequelae. Although the relationship between vitamin B1 deficiency and various symptoms is clear, findings indicating vitamin B1 deficiency in hospitalized patients in rural areas have not yet been elaborated. We previously demonstrated an association between appetite loss and vitamin B1 deficiency in hospitalized patients [11]. In this study, our objective was to measure vitamin B1 levels in patients with appetite loss or suspected vitamin B1 deficiency to identify additional predictors of this condition.

## Materials and methods

Study design

This single-center, cross-sectional study aimed to identify the predictors of vitamin B1 deficiency. Using data from electronic medical records, multivariate logistic regression was performed with vitamin B1 deficiency as the dependent variable and several covariates, including older age, sex, anemia, underweight, functional independent measure (FIM), Charlson Comorbidity Index (CCI), and polypharmacy.

Setting

In 2022, the total population of the rural area where the study was conducted was 35,738 (17,231 males and 18,507 females), and the percentage of urban residents aged 65 years and older was 40.27%. There was only one public hospital in the rural area. The rural hospital had 281 beds during the study period, including 155 acute, 48 general, 30 rehabilitation, and 48 chronic beds. Internal medicine patients were managed within the Department of Family Medicine through collaborative efforts with multiple healthcare professionals [12].

Participants

Study participants were selected from patients admitted to the hospital. The selection criteria included appetite loss, findings suggestive of vitamin B1 deficiency, and impaired consciousness (suspected Wernicke-Korsakoff syndrome). Data of patients treated from April 2020 to March 2022 were collected from the hospital's electronic medical records.

Data collection

In this study, vitamin B1 deficiency was used as the dependent variable. Vitamin B1 deficiency was defined as serum vitamin B1 level <20 µg/dL. Risk factors for vitamin B1 deficiency were determined based on previous studies and were evaluated as covariates; data for the covariates were also collected from electronic medical records. Covariates included age, sex, BMI, serum albumin, FIM, hemoglobin (Hb), CCI, and medications. Cases in which these data were not available were excluded from the study.

Statistical analysis

For continuous variables, the normality of the data was tested before applying statistical tests. Parametric and nonparametric data were analyzed using Student's t-test and Mann-Whitney U test. The chi-square test was employed to analyze categorical data. The following continuous variables were dichotomized for the logistic regression model: older age (>75 years or not), vitamin B1 deficiency (<20 µg/dL or not), hypoalbuminemia (<3.0 g/dL or not), anemia (Hb <12 g/dL or not), high CCI (>6 or not), and polypharmacy (medication ≥5 or not). Multivariate logistic regression analysis was performed to examine the association between vitamin B1 deficiency and these factors. Multivariate logistic regression models were constructed using all variables known to be associated with vitamin B1 deficiency, and the variables were found to be significant in the univariate regression models of vitamin B1 deficiency. All data analyses were carried out with Easy R, version 1.23 (R Foundation for Statistical Computing, Vienna, Austria). Statistical significance was set at p<0.05.

Ethical considerations

The hospital was assured that patient anonymity and the confidentiality of information would not be compromised. Information related to this study was posted on the hospital’s website without disclosing any patient details. In addition, the contact information of hospital personnel was posted on a website to address questions regarding this study. The goal of the study was explained to all patients, and informed consent was acquired. The Unnan City Hospital Clinical Ethics Committee approved the study protocol (no. 20190010).

## Results

Demographics of the participants

**Figure 1:**
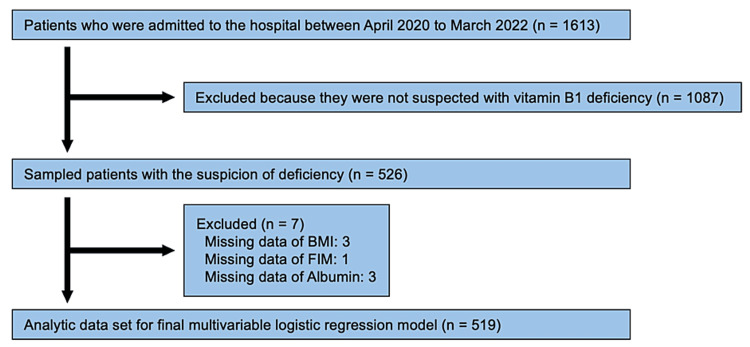
Flowchart depicting the selection of patients BMI: body mass index; FIM: functional independence measure

Figure 1 shows the flowchart illustrating the process of the selection of study participants; between April 2020 and March 2022, 1,613 patients were hospitalized. Based on the medical records, 526 patients with suspected vitamin B1 deficiency were included. Seven patients were excluded due to insufficient data on independent variables (Figure 1).

Of the 519 patients, 113 (21.8%) had vitamin B1 deficiency. The mean ages of the vitamin B1-deficient and non-deficient groups were 85.29 [standard deviation (SD): 7.62] and 84.08 (SD: 7.90) years, respectively. There was a significant difference in terms of age between the two groups (Table 1).

**Table 1:** Demographic data of the participants CCI: Charlson Comorbidity Index; FIM: functional independence measure; SD: standard deviation

Characteristics	Vitamin B1 deficiency			
	-	+		P-value
Age, mean (SD)	83.82 (10.62)	85.85 (8.31)		<0.05
Female sex, n (%)	233 (56.4)	63 (55.8)		0.915
Serum albumin, mean (SD)	3.47 (0.63)	3.36 (0.64)		0.115
Body mass index, mean (SD)	20.07 (3.84)	19.61 (3.36)		0.252
Hemoglobin, mean (SD)	10.9 (2.26)	12.0 (7.17)		0.088
CCI score (CCI ≥6), n (%)	174 (42.1)	55 (48.7)		0.239
Dementia	82 (19.9)	26 (23.0)		0.511
Diabetes	72 (17.4)	20 (17.7)		1
Kidney disease	34 (8.2)	9 (8.0)		1
Hepatic disease	23 (5.6)	6 (5.3)		1
Malignancy	92 (22.3)	25 (22.1)		1
FIM motor, mean (SD)	48.1 (30.4)	44.9 (29.4)		0.3
FIM cognition, mean (SD)	22.9 (11.6)	22.3 (11.5)		0.58
Medication, mean (SD)	6.5 (3.76)	6.1 (3.4)		0.27

Heart failure was the most common diagnosis, followed by bacterial pneumonia, urinary tract infections, stroke, and cancer (Table 2).

**Table 2:** Diagnosis among the participants

Rank	Diagnosis	Number	%		Number	Diagnosis	Number	%
1	Heart failure	76	14.4%		19	Angina	4	0.8%
2	Urinary tract infection	74	14.0%		20	Hyponatremia	4	0.8%
3	Brain stroke	55	10.4%		21	Hypothyroidism	4	0.8%
4	Pneumonia	24	4.5%		22	Ischemic colitis	4	0.8%
5	Aspiration pneumonia	23	4.3%		23	Unconsciousness	4	0.8%
6	Brain hemorrhage	17	3.2%		24	Brain tumor	3	0.6%
7	Sepsis	17	3.2%		25	Fever	3	0.6%
8	Cancer	16	3.0%		26	Gastric ulcer	3	0.6%
9	Epilepsy	14	2.6%		27	Hypoglycemia	3	0.6%
10	Syncope	13	2.5%		28	Interstitial pneumonia	3	0.6%
11	Appetite loss	11	2.1%		29	Iron deficiency anemia	3	0.6%
12	Pseudogout	9	1.7%		30	Medical complication	3	0.6%
13	Vitamin B1 deficiency	8	1.5%		31	Medication-induced	3	0.6%
14	Cellulitis	7	1.3%		32	Subdural hematoma	3	0.6%
15	Cholangitis	5	0.9%		33	Trauma	3	0.6%
16	Dehydration	5	0.9%		34	Wernicke encephalopathy	3	0.6%
17	Renal failure	5	0.9%			Others	96	18.1%

Association of vitamin B1 deficiency with influencing factors

Multivariate logistic regression analysis showed that anemia was significantly associated with vitamin B1 deficiency [adjusted odds ratio (AOR): 1.71, 95% confidence interval (CI): 1.07-2.73, p<0.05]. Older age, hypoalbuminemia, FIM, high CCI score, and polypharmacy were not significantly associated with vitamin B1 deficiency (Table 3).

**Table 3:** Results of the multivariate logistic regression model CCI: Charlson Comorbidity Index; FIM: Functional independence measure

Factor	Adjusted odds ratio	95% CI	P-value
Albumin	0.86	0.50–1.47	0.57
CCI	0.97	0.57–1.60	0.87
FIM motor	1.00	0.99–1.01	0.57
FIM cognition	0.99	0.97–1.02	0.66
Anemia	1.71	1.07–2.73	<0.05
Polypharmacy	0.81	0.54–1.32	0.47
Sex	0.87	0.56–1.35	0.53
Underweight	1.27	0.81–2.00	0.29
Older age	0.77	0.37–1.60	0.49

## Discussion

In this single-center retrospective study, we explored the factors predicting vitamin B1 deficiency among hospitalized patients in rural Japan. A significant difference in age was observed between the two cohorts. Age-related atrophy of the gastrointestinal mucosa can inhibit vitamin B1 absorption. Further studies are needed to elucidate the association between gastrointestinal mucosal atrophy and vitamin B1 absorption. Our data indicate a potential link between anemia and vitamin B1 deficiency in rural inpatients. Clinicians should consider vitamin B1 deficiency in the differential diagnosis when encountering rural inpatients with anemia.

Iron, vitamin B12, and folic acid are prevalent anemia-associated nutrients [13,14]. The gastric mucosa plays a pivotal role in the absorption of iron and vitamin B12 [15]. Anemia might indicate a compromised physiological function of the gastrointestinal system, possibly leading to reduced vitamin B1 absorption. Future research should assess the gastrointestinal function in vitamin B1-deficient patients to more accurately detect its relationship with anemia. Heavy alcohol consumption, which depletes vitamin B1 levels, contributes to Wernicke-Korsakoff syndrome. Alcohol impedes vitamin B12 absorption, further exacerbating anemia [16]. The dietary patterns of alcoholics, which are often marked by reduced intake, can further diminish vitamin B12 and iron levels. Future studies are needed to delineate the relationship between vitamin B1 levels, alcohol consumption habits, and anemia. Another aspect to consider is thiamine-responsive megaloblastic anemia (TRMA) syndrome [17], an autosomal recessive disorder stemming from anomalies in the vitamin B1 transporter (SLC16A2) within the gastrointestinal system [18]. While TRMA generally manifests during childhood with diabetes and progressive sensorineural hearing loss, our study mainly included patients with atypical, late-onset TRMA.

Vitamin B1 deficiency manifests in diverse clinical scenarios and should be managed through comprehensive care and interprofessional collaboration. Loop diuretics, commonly prescribed for heart failure, can exacerbate this deficiency, whereas vitamin B1 supplementation may offer therapeutic benefits [19]. Conditions such as Wernicke's encephalopathy, often induced by alcohol or other etiologies, lead to cerebral vitamin B1 deficits and produce a spectrum of neuropsychiatric symptoms [20]. Korsakoff's syndrome, which stems from untreated Wernicke's encephalopathy, presents with a plethora of neurological and psychiatric anomalies [20]. For hospitalized patients with ambiguous symptoms such as heart failure or neuropsychiatric presentations, assessing vitamin B1 levels is paramount. Anemia is prevalent and significantly associated with hospital readmission among older patients [21]. Upon suspicion of vitamin B1 deficiency with anemia, especially in older patients, intravenous vitamin B1 should be administered, or a gastroenterology consultation should be sought for potential malabsorption. Immediate vitamin B1 supplementation is crucial for older adults with unexplained psychiatric symptoms or diminished appetite. Moreover, the readmission rates among rural community hospitals can be improved through interprofessional collaboration [22]. Comprehensive care with interprofessional collaboration should be implemented in rural medical care.

This study has some limitations. Primarily, there is a potential selection bias, as our participants, chosen based on appetite loss or signs indicating vitamin B1 deficiency, were not randomized. Future studies should adopt a prospective randomized participant selection approach. Secondly, local dietary habits characterized by limited red meat consumption among older adults may have affected our results. Replicating this study in various different geographical settings is crucial, considering the paucity of research on vitamin B1 deficiency elsewhere. Third, the nutritional regimen of long-term hospitalized patients, provided by the hospital diet, might have rectified the vitamin B1 deficiency before assessment. Finally, the behavioral nuances of our participants, who were primarily hospitalized during the coronavirus disease 2019 (COVID-19) pandemic and influenced by Japan's proactive preventive measures, might have deviated from typical patterns.

## Conclusions

This retrospective study of hospitalized patients in rural Japan revealed a significant association between anemia and vitamin B1 deficiency. Although a significant age difference was observed between the vitamin B1-deficient and non-deficient groups, other variables, such as albumin level, FIM, CCI, and medication count, did not significantly correlate with vitamin B1 deficiency. Therefore, clinicians should be mindful of the possibility of vitamin B1 deficiency in rural inpatients with anemia.
